# Glutathione Upregulates the Expression of K_ATP_ Channels and Vasorelaxation Responses and Inhibits mPTP Opening and Oxidative Stress in the Heart Mitochondria of Old Rats

**DOI:** 10.1155/2023/3562847

**Published:** 2023-05-24

**Authors:** Ruslan Strutynskyi, Nataliіa Strutynska, Lidiia Mys, Yulia Goshovska, Yuliia Korkach, Raisa Fedichkina, Iryna Okhai, Vladyslav Strutynskyi, Vadym Sagach

**Affiliations:** ^1^Department of General and Molecular Pathophysiology, Bogomoletz Institute of Physiology, National Academy of Sciences of Ukraine, 4, Bogomoletz Str., Kyiv 01024, Ukraine; ^2^Department of Blood Circulation, Bogomoletz Institute of Physiology, National Academy of Sciences of Ukraine, 4, Bogomoletz Str., Kyiv 01024, Ukraine; ^3^Department of Immunophysiology, Bogomoletz Institute of Physiology, National Academy of Sciences of Ukraine, 4, Bogomoletz Str., Kyiv 01024, Ukraine

## Abstract

**Background:**

In the present work, we investigated the effect of exogenous glutathione in old rats on the expression of ATP-sensitive potassium (K_ATP_) channels, the mitochondrial permeability transition pore (mPTP) opening in the heart, and the vasorelaxation responses of isolated aortic rings to activation of K_ATP_ channels.

**Methods:**

Experiments were performed on adult (6 months) and old (24 months) male Wistar rats, which were divided into three groups: adult, old, and glutathione-treated old rats. Glutathione was injected intraperitoneally at a dose of 52 mg/kg 1 hour before the studies. The mRNA expression of K_ATP_ channels was determined using reverse transcription and real-time polymerase chain reaction analysis. The effect of glutathione administration on mPTP opening, relaxation responses of isolated aortic rings, and oxidative stress markers was studied.

**Results:**

It was shown that the expression levels of Kir6.1, Kir6.2, and SUR1 subunits of K_ATP_ channels and levels of reduced glutathione were significantly increased in glutathione-treated old rats (by 8.3, 2.8, 13.1, and 1.5-fold, respectively), whereas the levels of oxidative stress markers (hydrogen peroxide, diene conjugates, malondialdehyde, and rate of superoxide generation) in heart mitochondria and mPTP opening were significantly reduced. Relaxation of aortic rings was significantly increased in response to the actions of K_ATP_ channel openers flocalin and pinacidil in glutathione-treated animals, which was prevented by glibenclamide.

**Conclusions:**

Thus, the administration of exogenous glutathione to old rats resulted in a significant increase in the expression levels of the Kir6.1, Kir6.2, and SUR1 subunits of K_ATP_ channels and a decrease in oxidative stress. This was accompanied by inhibition of mPTP opening and enhancement of vasorelaxation responses to activation of K_ATP_ channels.

## 1. Introduction

Glutathione (*γ*-glutamyl-cysteinyl-glycine; GSH) and the system of sarcolemmal and mitochondrial ATP-sensitive potassium (K_ATP_) channels are powerful defense mechanisms. Glutathione is a low-molecular-weight thiol that can be in reduced (GSH) and oxidized (glutathione disulfide, GSSG) state and participates in the maintenance of cellular redox homeostasis [1, 2]. In its reduced form, it has strong antioxidant properties, is an electron donor, and plays an important role in protecting against oxidative stress. Glutathione also supports the efficient functioning of protein systems, especially the mitochondrial electron transport chain, ATPase activity, ion channels, transporters, etc., and GSH/GSSG imbalance can be associated with pathological changes [1–3]. K_ATP_ channels are one of the main endogenous mechanisms of cell protection when its energy resources are reduced. They are considered to be the central metabolic sensor of the cell regarding its energy supply [4]. These channels play a unique role in the synchronization of cellular metabolism and electrical activity, regulation of potential-dependent membrane functions, and maintenance of hormonal homeostasis, and dysfunction of K_ATP_ channels may contribute to the pathogenesis [5]. At the same time, with age, an imbalance of these protective systems can occur, both a violation of the intracellular GSH/GSSG ratio and a decrease in the expression of K_ATP_ channels, which can be accompanied by a decrease in resistance to pathological processes and by an increase in sensitivity to pathological factors. In particular, oxidative and nitrosative stress may increase with age, and dysfunction of mitochondrial mechanisms may occur, including with the formation of a nonselective mega channel—the mitochondrial permeability transition pore (mPTP) in the mode of high conductivity (which is the cause of the development of many pathological conditions of the body), may the resistance to ischemia decreases, the tendency to arterial hypertension increases, etc. [1, 6–10]. One of the ways to prevent these dysfunctions is to increase the ratio of GSH/GSSG and the expression of K_ATP_ channels in the tissue. Recently, we showed that course administration of pyridoxal-5-phosphate, a cofactor of H_2_S-synthesizing enzymes, in old rats increased not only the level of hydrogen sulfide in tissues but also the expression of Kir6.1 and SUR2 subunits of K_ATP_ channels, which was accompanied by a decrease in oxidative stress and a decrease in reperfusion dysfunction heart during ischemia-reperfusion [9]. In general, the K_ATP_ channel has an octameric structure and consists of 4 pore-forming Kir6.x subunits and 4 regulatory SUR subunits surrounding the pore. Three types of SUR are known: SUR1, SUR2A, and SUR2B, and two Kir6.x types: Kir6.1 and Kir6.2. Their combinations determine the properties of the channel and correspond to certain tissues. In the cardiovascular system, Kir6.1 is mainly prevalent in the vascular tissue, and Kir6.2 is prevalent in the myocardium. Together with SUR2, these Kir6.x form a full-fledged K_ATP_ channel in the vascular system and the heart, respectively. It has also been shown that H_2_S donors can increase the expression of SUR2B and Kir6.1 subunits of vascular-type K_ATP_ channels in spontaneously hypertensive rats, and this was accompanied by a reduction in blood pressure [11]. However, so far little attention has been paid to the study of the interaction of such powerful endogenous defense mechanisms as K_ATP_ channels and glutathione. And if there is still information about improving the glutathione system functioning at activation of K_ATP_ channels and about changing the activity of K_ATP_ channels by glutathione, then, the mechanism of modulation of the expression of K_ATP_ channels by GSH is shown for the first time by us [12–14]. The aim of this work was to study the possible influence of exogenous glutathione administration on regulating the expression of K_ATP_ channels, mPTP opening in the heart, and vasorelaxation of isolated aortic rings upon activation of K_ATP_ channels in old rats.

## 2. Materials and Methods

### 2.1. Animals

All procedures were conducted in accordance with the Directive 2010/63/EU of the European Parliament and of the Council on the protection of animals used for scientific purposes (22.09.2010). The experimental protocols were approved by the Biomedical Ethics Committee of the Bogomoletz Institute of Physiology National Academy of Sciences of Ukraine, Ukraine (No.2/21, June 16, 2021). The experiments were performed on adult (6 months old, body mass 220-250 g) and old (22-24 months old, body mass 350-450 g) male Wistar rats. Animals were housed in a neutral temperature environment (22°C ± 2°C) on a natural day-to-night cycle with free access to water and on a standard diet. Rats were divided into three groups: adult, old, and glutathione treatment old animals. In each series of experiments, from 7 to 15 animals were used. Exogenous GSH was diluted in the physiological solution and injected intraperitoneally at a dose of 52 mg/kg 1 hour before test preparations.

### 2.2. RNA Isolation and Real-Time Polymerase Chain Reaction (PCR) Analysis

The total RNA was isolated from the heart tissue using Tri reagent (Sigma-Aldrich). The concentration and purity of total RNA were determined using a NanoDrop spectrophotometer ND1000 (NanoDrop Technologies Inc, USA). Reverse transcription was performed using the RevertAid First Strand cDNA Synthesis Kit (Thermo Fisher Scientific, USA). For KCNJ8, KCNJ11, ABCC8, and ABCC9 genes encoding Kir6.1, Kir6.2, SUR1, and SUR2 subunits of K_ATP_ channels, respectively, and beta-actin, RT-PCR amplification reaction was performed in a volume of 10 *μ*mol/L containing 0.1 *μ*mol/L forward/reverse primers, 5 *μ*mol/L SYBR-Green PCR Master Mix, and 1 *μ*mol/L cDNA. The primer sequences used for RT-PCR are presented in Table 1. PCR was performed for 50 cycles of 10 min at 95°C, 15 sec at 95°C, and 60 sec at 60°C using 7500 Fast Real-Time PCR (Applied Biosystems, USA). The threshold cycle (Ct) was automatically calculated by the instrument software. Calculations were standardized by the housekeeping gene, beta-actin. Data analysis was performed with 7500 Fast Real-Time PCR Software.

### 2.3. Registration of Mitochondrial Swelling in the Heart of Rats

Hearts were removed from decapitated rats and washed with a cold 0.9% solution of KCl (4°C). Mitochondria were isolated by differential centrifugation, and protein content was determined in organelle suspension by the method of Lowry. mPTP opening was investigated by spectrophotometric registration of the swelling of mitochondria isolated from the rat heart. For this purpose, mitochondria were placed in the incubation medium of isotonic composition (mmol/L): KCl–120, Tris-HCl–25, KH_2_PO_4_–3, sodium succinate–5, pH 7.4, and a decrease in the optical density in mitochondria suspension was recorded at *λ* = 520 nm for 15 min of the mitochondria swelling. Protein concentration was 0.4 mg/mL. As a control, mitochondrial suspension was used in the incubation medium in the absence of an inducer, with the following registration of the optical density for 15 min. The amplitude (A) of spontaneous (control) and maximum swelling of the organelle suspension in Ca^2+^-free medium and in the conditions of pore-formation activation under the action of PTP inducer of swelling of calcium ions was calculated as the difference in optical density values from 1 to 15 minutes. Cyclosporin A (Cs A), flocalin, and 5-hydroxydecanoate (5-HD) were preincubated in the medium with mitochondria for 5 minutes before adding the calcium inducer. DL-buthionine-S,R-sulfoximine (BSO) at a dose of 100 *μ*mol/kg was injected intraperitoneally 30 min before decapitation of the animals. The results were processed by methods of variation statistics using the program Origin 7.0 (“Microcall Inc”, USA).

### 2.4. A Study of the Relaxation Responses of Isolated Aortic Rings

The experiments were performed on isolated aortic rings of rats perfused at a temperature of 37°С normal Krebs solution. All tests were performed in isometric mode at the initial set voltage at which they generated maximum force in response to noradrenaline infusion (10 *μ*mol/L). The temperature of the solution in the experimental chamber (37°C ± 0.5°C) was maintained using an automatic thermostat KISS 208B (Huber). The working solution was saturated with oxygen using a gas mixture containing 95% O_2_ and 5% CO_2_. Before measurement, the vascular strips fixed in the experimental chamber were kept for 60 min in a normal Krebs solution of the following composition (in mmol/L): NaCl-120.4; KCl-5.9; NaHCO_3_-15.5; NaH_2_PO_4_-1.2; MgCl_2_-1.2; CaCl_2_-2.5; glucose-11.5. Studies of vasodilatory effects were performed against the background of increased tone of preparations of the aorta, which was obtained by perfusion of norepinephrine (10 *μ*mol/L). For activation of K_ATP_ channels, pharmacological opener of K_ATP_ channel flocalin in doses from 0.01 to 100 *μ*mol/L is used [15, 16]. The nature of vasodilatory responses was determined using a specific inhibitor of K_ATP_ channel glibenclamide, which in dose, 10 *μ*mol/L was injected into a perfusion solution 5 minutes before injecting flocalin. To determine the state of the endothelial layer of vessels, we studied endothelium-dependent reactions of vascular rings with the help of acetylcholine, which was injected into the perfusion solution in dose 1 *μ*mol/L.

### 2.5. Determination of the Oxidative Stress Markers and Glutathione Levels in Heart Tissue

Biochemical indicators of oxidative stress such as the rate of formation of superoxide (•O_2_^−^) and hydrogen peroxide pools (H_2_O_2_) were measured in the suspension of cardiac mitochondria. Pools of diene conjugates (DС) and malondialdehyde (MDA) were measured as markers of lipid peroxidation. The methods used to assess oxidative stress are described in detail in our previous work [6]. The protein content in the samples of heart tissue homogenates was determined by the Lowry method.

The measurements of GSSG and GSH were performed in the heart homogenates with Ellman's reagent [17]. Measurements were performed using a Biosan HiPo MPP-96 microplate reader (Lithuania). 60 *μ*L of 500 unit glutathione reductase in KPE solution (1 : 150), 60 *μ*L 0.8 mmol/L cofactor *β*-NADPH, and 60 *μ*L 1.68 mmol/L dithiobis nitrobenzoic acid were added to initiate the reaction. Optical density was measured immediately and for 2 minutes each 30 seconds at 405 nm. The data were presented in moles per milligram of the examined tissue.

### 2.6. Statistical Analysis

The data were expressed as mean ± SEM (standard error of the mean). The Shapiro-Wilk test was used to evaluate the normality of distribution of data in each group. Comparison between groups was made using one-way analysis of variance (ANOVA) followed by *post hoc* Tukey's HSD test or the nonparametric Kruskal-Wallis test for multiple independent samples with *post hoc* test by the methods of Conover. *P* < 0.05 was assumed as statistically significant.

## 3. Results

### 3.1. Effect of GSH Administration on Kir6.1, Kir6.2, SUR1, and SUR2 Expression

**Figure 1:**
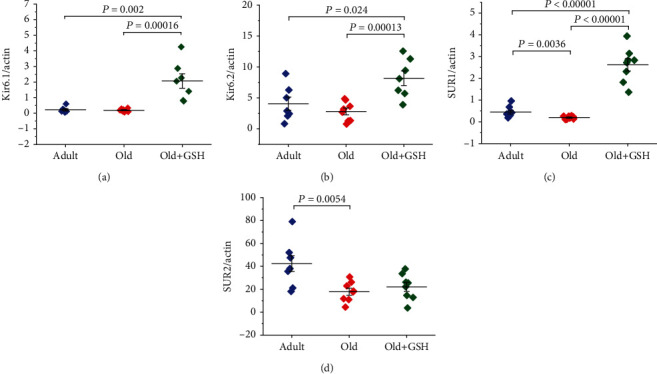
mRNA levels of genes encoding Kir6.1 (a), Kir6.2 (b), SUR1 (c), and SUR2 (d) subunits of K_ATP_ channels in the heart of adult (6 months) and old (24 months) male Wistar rats and glutathione pretreatment male old rats. Data are represented as mean ± SEM. For adult rats, *n* = 8; for old rats, *n* = 11; for old glutathione-treated rats, *n* = 7. GSH: glutathione. The figure shows a significantly lower expression of mRNA of genes encoding SUR1 (c) and SUR2 (d) regulatory subunits of K_ATP_ channels in the heart of old rats compared to adult animals. In glutathione administration experiments, the expression levels of Kir6.1 (a), Kir6.2 (b), and SUR1 (c) in the heart of glutathione-treated old rats were significantly higher compared to untreated old animals.

No significant differences were found in the levels of mRNA expression of pore-forming Kir6.1 subunits of predominantly vascular-type (Figure 1(a)) and pore-forming Kir6.2 subunits of predominantly cardiac-type (Figure 1(b)) sarcolemmal K_ATP_ channels in the heart of adult and old rats. GSH administration of old rats significantly increased the expression of both Kir6.1 (8.28-fold, *P* < 0.0002) and Kir6.2 (2.84-fold, *P* < 0.0002) subunits of K_ATP_ channels (Figures 1(a) and 1(b)). Determination of the mRNA expression levels of the regulatory subunits SUR1 and SUR2 of K_ATP_ channels in the heart of adult and aged rats revealed a significant decrease in their expression in the heart of aged animals, namely, for SUR1 by 2.25-fold (*P* = 0.0036) and for SUR2 by 2.37-fold (*P* = 0.0054) (Figures 1(c) and 1(d)). Glutathione administration of old rats significantly increased (13.1-fold, *P* < 0.0001) the mRNA expression levels of SUR1 subunits, predominantly the mitochondrial-type K_ATP_ channels in the heart tissue (Figure 1(c)). At the same time, GSH administration in old rats practically did not affect the expression levels of SUR2 regulatory subunits of the K_ATP_ channels (Figure 1(d)).

Thereby, the levels of mRNA expression of SUR1 and SUR2 regulatory subunits of K_ATP_ channels were significantly lower in the heart of old rats compared with adult animals (Figure 1). In glutathione administration experiments, the expression levels of Kir6.1, Kir6.2, and SUR1 were significantly higher in the heart of glutathione-treated old rats compared with untreated old animals.

### 3.2. Effect of GSH Administration on Relaxation Responses of Isolated Aortic Rings

**Figure 2:**
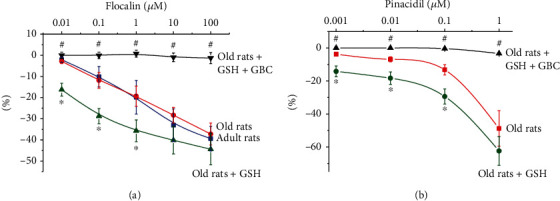
Vasodilatory effects of openers of K_ATP_ channels of flocalin (a) and pinacidil (b) in experiments on isolated aortic rings of adult (6 months) and old (24 months) male Wistar rats, as well as old male glutathione-treated animals. GSH: glutathione. GBC: glibenclamide. Data are represented as mean ± SEM. For adult rats, *n* = 11; for old rats, *n* = 15; for old glutathione-treated rats, *n* = 15. ^∗^*P* < 0.05 compared to old rats. ^#^*P* < 0.05 compared to old glutathione-treated rats. The figure shows that the relaxation of vascular rings in response to the activation of K_ATP_ channels by flocalin did not differ in adult and old rats (a). At the same time, the administration of glutathione to old rats significantly enhances the vasodilatory reactions of the K_ATP_ channel openers floсalin (a) and pinacidil (b). The vasodilator effects of floсalin and pinacidil were prevented by the K_ATP_ channel inhibitor glibenclamide.

Relaxation of the ring preparations of the thoracic aorta of both groups of old rats, both treated and not treated with GSH, in response to the administration of the pharmacological openers of K_ATP_ channel flocalin (in doses of 0.01-100 *μ*mol/L) and pinacidil (in doses of 0.001-1 *μ*mol/L) was dose-dependent (Figure 2). Preperfusion of vascular rings for 5 min with a specific inhibitor of K_ATP_ channels, glibenclamide at a dose of 10 mmol/L, prevented the vasodilatory effects of flocalin and pinacidil (Figure 2), which may indicate that these relaxation vascular reactions occur due to the activation of K_ATP_ channels of cell membranes. The vasodilatory effects of flocalin on the rings of the aorta of adult and old rats practically did not differ (Figure 2(a)). The relaxation responses of the vascular rings of old rat GSH-treated in response to the effect of different doses of flocalin differed significantly from the reactions of untreated control animals (Figure 2(a)). Similar differences in the value of the relaxation of ring preparations of the aorta of old rats treated and untreated with glutathione were also observed under the action of pinacidil (Figure 2(b)). Administration of GSH significantly increased the amplitude of the vasodilatory effects of the pharmacological opener of K_ATP_ channel flocalin, namely, by 5.67 times (*P* = 0.006), 2.42 times (*P* = 0.005), 1.85 times (*P* = 0.034) and by 1, 42, and 1.2 times at flocalin doses of 0.01, 0.1, 1, 10, and 100 *μ*mol/L, respectively (Figure 2(a)). The vasodilator effects of pinacidil in doses of 0.001, 0.01, 0.1, and 1 *μ*mol/L in GSH-treated old animals were greater than on aorta preparations of untreated animals by 3.75 times (*P* = 0.0056), 2.68 times (*P* = 0.0125), and 2.22 times (*P* = 0.0127) and by almost a third, respectively (Figure 2(b)).

Thereby, in experiments with isolated aortic rings, it was shown that administration of glutathione to old rats significantly enhanced the vasodilatory responses to the K_ATP_ channel openers pinacidil and flocalin. The effects were prevented by the inhibitor of these channels, glibenclamide. There was no difference between adult and old rats in the relaxation of vascular rings in response to the activation of K_ATP_ channels.

### 3.3. Ca^2+^-Induced Opening of mPTP in the Heart of GSH-Treated Old Rats

**Figure 3:**
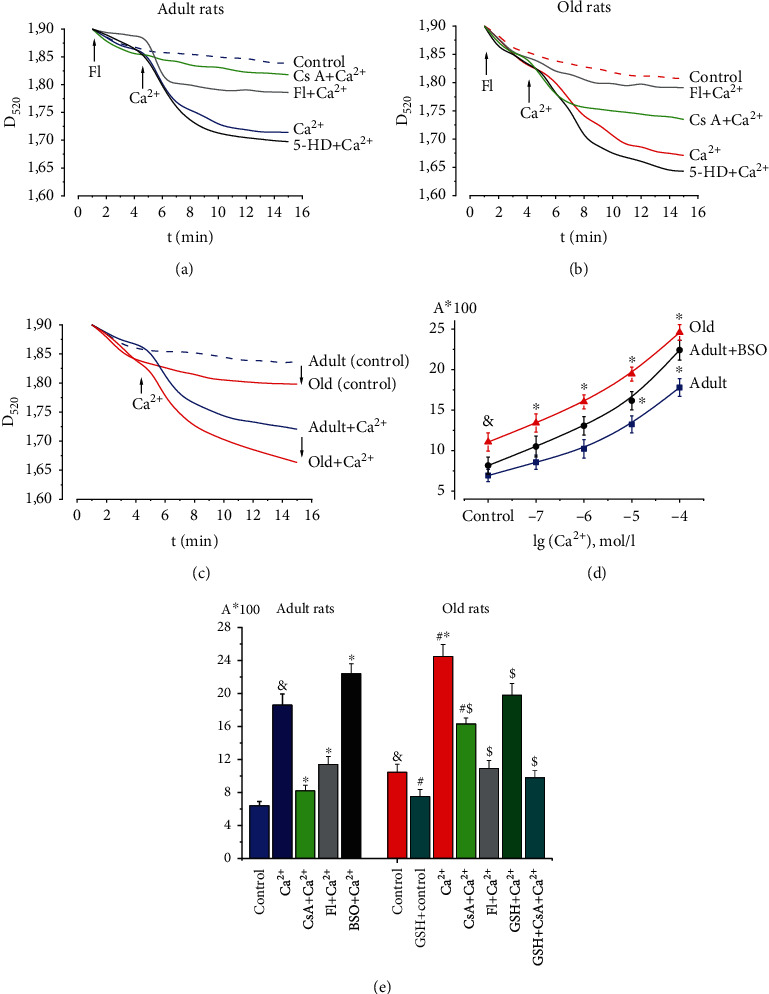
Effects of glutathione, modulators of glutathione synthesis, and K_ATP_ channel activity on the Ca^2+^-induced mPTP opening in heart of adult (6 months) and old (24 months) rats. (a) Effect of K_ATP_ channel modulators flocalin (10 *μ*mol/L) and 5-HD (100 *μ*mol/L) on Ca^2+^-induced swelling of heart mitochondria of adult rats (typical native curves). (b) The action of K_ATP_ channel modulators flocalin (100 *μ*mol/L) and 5-HD (100 *μ*mol/L) on Ca^2+^-induced swelling of heart mitochondria of old rats (typical native curves). (c) Comparison of the amplitude of control (without calcium) and calcium-dependent swelling of heart mitochondria of adult and old rats. (d) The sensitivity of mPTP to calcium in the heart of adult and old rats, as well as under the conditions of action of the inhibitor of glutathione synthesis BSO in the heart of adult rats. (e) Influence of glutathione and its synthesis inhibitor BSO, K_ATP_ channel opener flocalin, and mPTP inhibitor Cs A on the amplitude of Ca^2+^-induced swelling (Ca^2+^, 100 *μ*mol/L) of heart mitochondria of adult and old rats. Glutathione and BSO were applied in vivo. GSH: glutathione; Cs A: classical mPTP inhibitor cyclosporin A; BSO: an inhibitor of glutathione synthesis DL-buthionine-S,R-sulfoximine; Fl: opener of K_ATP_ channels flocalin; 5-HD: an inhibitor of mitochondrial K_ATP_ channels 5-hydroxydecanoate. Data are represented as mean ± SEM. For adult rats, *n* = 11; for old rats, *n* = 11; for old glutathione-treated rats, *n* = 12. ^&^*P* < 0.05 compared to control adult rats (Ca^2+^-free medium); ^#^*P* < 0.05 compared to control old rats (Ca^2+^-free medium); ^∗^*P* < 0.05 compared to Ca^2+^-induced swelling of heart mitochondria of adult rats; ^$^*P* < 0.05 compared to Ca^2+^-induced swelling of heart mitochondria of old rats. The use of exogenous glutathione and its synthesis inhibitor BSO and K_ATP_ channel activity modulators flocalin and 5-HD in studies allowed us to draw conclusions about the regulation of pore formation with the help of glutathione, as well as about the effect of activation of K_ATP_ channels on inhibition of mPTP. Increased sensitivity of mPTP to its calcium inducer was shown in old animals. The mitoprotective effect of exogenous glutathione in its reduced form was manifested by partial inhibition of Ca^2+^-induced mPTP opening.

Figure 3(a) shows typical kinetic curves of spontaneous (control in calcium-free medium, *A*_cntrl_) and calcium-induced (*A*_Ca_) swelling of heart mitochondria in adult rats. Calcium loading of mitochondria resulted in high-amplitude swelling, which was almost completely prevented by the classical mPTP inhibitor Cs A at a concentration of 10 *μ*mol/L (Figures 3(a) and 3(e)). Mitochondrial swelling under Ca^2+^ action in the presence of Cs A was near to control as in a calcium-free medium (Figures 3(a) and 3(e)). This may be evidence that mitochondrial swelling is indeed due to mPTP opening. Under the conditions of preincubation of mitochondria with the K_ATP_ channel opener flocalin, there was a dose-dependent decrease in the amplitude of calcium-induced swelling of the organelles. The half-maximal effect occurred at a flocalin concentration of 50 *μ*mol/L. At 10 *μ*mol/L flocalin dose, Ca^2+^-induced mitochondrial swelling was significantly reduced by 59.0% (*P* < 0.05) (Figure 3(e)). The difference between *A*_Ca_ and *A*_cntrl_ was taken as 100%. Preincubation of isolated mitochondria with the inhibitor of mitochondrial K_ATP_ channel 5-HD (100 *μ*mol/L) had practically no effect on the swelling of the organelles under the conditions of Ca^2+^-loading (Figure 3(a)). Amplitudes of control and calcium-dependent swelling of heart mitochondria of old rats, which was recorded at the 15th minute, significantly exceeded similar indicators in adult animals by 1.8 times (*P* < 0.05) and by a third (*P* < 0.05), respectively (Figures 3(c)–3(e)). This suggests an increased sensitivity of the mPTP to its inducer calcium in old animals. Under conditions of calcium loading of mitochondria, high-amplitude swelling in old animals was partially suppressed by Cs A (Figures 3(b) and 3(e)). This suggests the existence of a Cs A-insensitive mPTP component in the aging heart. The K_ATP_ channel opener flocalin (100 *μ*mol/L) almost completely (by 91.3%, *P* < 0.05) inhibited Ca^2+^-induced swelling of heart mitochondria of old animals (Figures 3(b) and 3(e)). Preincubation of isolated mitochondria with the mitochondrial K_ATP_ channel inhibitor 5-HD (100 *μ*mol/L) did not significantly change the amplitude of organelle swelling under Ca^2+^ loading conditions (Figure 3(b)).

It was shown that the sensitivity of mPTP to Ca^2+^ at concentrations of 10 and 100 *μ*mol/L was reliably increased by 22.1% (*P* < 0.05) and 31.1% (*P* < 0.05), respectively, under conditions of short-term inhibition of glutathione biosynthesis in vivo by DL-buthionine-S,R-sulfoximine (BSO) (100 *μ*mol/kg) in adult rats (Figure 3(d)). This may indicate the importance of glutathione in the maintenance of mitochondrial redox status and regulation of mitochondrial nonspecific permeability (mPTP opening). The results of studying the effect of exogenous glutathione on mPTP opening in heart mitochondria of old rats are presented in Figure 3(e). It was shown that in glutathione-treated old rats compared to untreated animals, both in a calcium-free environment and under the influence of calcium, the swelling amplitudes of mitochondria were significantly smaller by 34.5% (*P* < 0.05) and 36.0% *P* < 0.05), respectively, and approached the swelling values in adult animals (Figure 3(e)). Thus, glutathione in its reduced form prevented the increase in spontaneous (*A*_cntrl_) and Ca^2+^-induced (*A*_Ca_) swelling of mitochondria, indicating the inhibition of pore formation in the heart of treated old animals (Figure 3(e)). The mPTP inhibitor Cs A was reduced by 62.8% (*P* < 0.05) Ca^2+^-induced swelling of heart mitochondria in old animals (Figure 3(e)). At the same time, in the background of glutathione, Cs A practically completely prevented mPTP formation in old rats, similar to its effects in adult animals (Figure 3(e)).

Therefore, the use in experimental studies of exogenous glutathione and its synthesis inhibitor BSO, K_ATP_ channel activity modulators (flocalin and 5-HD) allowed us to draw conclusions about the regulation of pore formation using glutathione and about the effect of activating K_ATP_ channels on inhibiting mPTP. Increased sensitivity of mPTP to its calcium inducer was demonstrated in old animals. The mitoprotective effect of exogenous glutathione in its reduced form was manifested by partial inhibition of Ca^2+^-induced mPTP opening.

### 3.4. Oxidative Stress Markers in Heart Mitochondria in GSH-Treated Old Rats

Determination of biochemical indicators of oxidative stress in heart mitochondria of old animals showed a significant increase compared to adult rats (Table 2). Namely, the rate of ^•^О_2_^−^ generation and H_2_O_2_ levels in heart mitochondria of old animals was increased by 3.9 times and almost twice, respectively. At the same time, the rate of generation of ^•^О_2_^−^ in GSH-treated old animals was decreased by 2.8 times compared with untreated animals (*P* < 0.05). The levels of H_2_O_2_ in heart mitochondria of old animals after administration of GSH were reduced by more than half, and their value approached the level of adult animals (Table 2). Also, the levels of lipid peroxidation products, both primary (DC) and final (MDA) products, significantly increased in old animals, namely, they were higher by more than four and 2.5 times, respectively, compared to adult animals. At the same time, in GSH-treated old animals, the levels of these lipid peroxidation products were significantly reduced: twice (*P* < 0.05) for DC and 1.6 times (*P* < 0.05) for MDA (Table 2).

Thus, the administration of glutathione to old rats significantly reduced biochemical markers of oxidative stress, which were substantively elevated in old animals compared to adults.

### 3.5. Levels of the Total, Reduced, and Oxidized Glutathione in the Heart Tissue

The results of studies of levels of total, reduced, and oxidized glutathione in the heart tissue are presented in Table 2. In our studies, the total level of glutathione in the heart tissue of adults and old rats practically did not differ. At the same time, there was a change in the balance between GSH and GSSG in old animals compared to adults. Thus, GSH levels in old rats were reduced by 15.1% (*P* = 0.023), while GSSG levels, on the contrary, increased by 19.8%. Administration of exogenous glutathione to old rats predictably increased total glutathione levels in heart tissue (by 40.3%, *P* = 0.0034). Analysis of the data showed that this increase in total content in glutathione-treated rats was largely due to an increase in GSH levels by 62.8% (*P* = 0.0034). At the same time, GSSG levels in the heart tissue of treated and untreated old rats with glutathione were not significantly different (Table 2).

Consequently, there is an imbalance between GSH and GSSG in the heart tissue of old rats compared to adult animals, toward a decrease in the reduced form of glutathione. The administration of exogenous glutathione to old rats resulted in a significant increase in both the total level of glutathione in the heart tissue and its reduced form.

## 4. Discussion

The system of K_ATP_ channels is one of the defense mechanisms controlling the energy balance, and by limiting the calcium entry into the cell, it can inhibit the processes of excitation and metabolic activity, in which the conditions of energy deficit preserve ATP reserves and prevent cell death. The protective mechanisms of K_ATP_ channels are realized by complex mechanisms, ranging from changes at the molecular level to changes at the systemic level, in particular in cardiohemodynamics. These protective mechanisms include both membrane stabilization with preserving sarcolemmal integrity and cell organelle structure (in particular mitochondria) during ischemia and reactions of the vascular system and heart. Positive vascular responses in ischemia-reperfusion can be considered a moderate decrease in blood pressure and prevention of an increase in the resistance of coronary vessels during reperfusion [18, 19]. It is an important relative preservation of the contractility of the ischemic myocardium during reperfusion [18, 19]. Activation of K_ATP_ channels during ischemia increases the protective constitutive synthesis of nitric oxide and suppresses the formation of oxygen and nitrogen free radicals [20, 21]. Probably that the reduction of oxidative and nitrosative stress occurs due to a decrease in the level of calcium in the cytoplasm, inhibition of metabolic processes, and the activity of enzymes of the tricarboxylic acid cycle [20, 22]. Activation of K_ATP_ channels during ischemia also maintains at a high level the activity of enzymes of the antioxidant system catalase and superoxide dismutase, prevents mPTP opening, and suppresses apoptosis and necrosis of cardiomyocytes [23, 24].

In experiments with ischemia-reperfusion and experimental myocardial infarction, it was shown that with a higher density of K_ATP_ channels on the membrane, their protective effect becomes more powerful, and increasing the expression of sarcolemmal K_ATP_ channels in cardiomyocytes results in an ischemia-resistant phenotype of the heart [25–28]. A decrease in expression of the Kir6.1 and SUR2B subunits of vascular-type K_ATP_ channels is often accompanied by arterial hypertension [29], whereas an increase in expression of these subunits is associated with an increase in vasodilator responses to pharmacological openers of these channels and a decrease in arterial pressure [11]. Pharmacological activators are used to activate K_ATP_ channels, but the strength of these protective effects depends on the density of K_ATP_ channels on cell membranes. Therefore, it seems appropriate to search for new triggers for upregulation of the expression of K_ATP_ channels in cell membranes in order to increase the body's endogenous resistance to the action of pathological factors.

In our experiments, intraperitoneal administration of exogenous GSH to old rats was associated with stimulation of the expression of three subunits of K_ATP_ channels, namely, the pore-forming Kir6.1 and Kir6.2 subunits of sarcolemmal-type channels and the regulatory subunit SUR1 of mitochondrial-type channels. At the same time, when glutathione was administered to old rats, the expression of cardio-specific SUR2 receptor K_ATP_ channels of sarcolemmal membranes of cardiomyocytes remained practically unchanged. And all of this is reflected in the function of the cardiovascular system and the readiness for protection against pathological factors. It is an increase in blood pressure with arterial hypertension or ischemic factors. According to the work of Sun et al. [11], an increase in the expression of vascular-type K_ATP_ channels with their reduced expression in spontaneously hypertensive rats was accompanied by an increase in vasodilator reactions in response to the opener of these channels, pinacidil. In our studies, the expression of Kir6.1 subunits of vascular-type K_ATP_ channels was significantly increased in glutathione-treated old animals, which was associated with a significant increase in the relaxation of isolated aortic rings under the action of the K_ATP_ channel openers flocalin and pinacidil, compared with relaxation in untreated animals. Thus, increasing the expression of Kir6.1 in GSH-treated old rats can probably increase the dilatation ability of vascular.

In general, increased expression of the pore-forming subunits Kir6.1 and Kir6.2 of vascular- and cardiac-type K_ATP_ channels, respectively, and the regulatory subunit SUR1 of mitochondrial-type K_ATP_ channels in GSH-treated old rats should contribute to the strengthening of endogenous protective mechanisms. In particular, increased swelling of heart mitochondrial in old rats compared with adults was significantly reduced in GSH-treated animals. It is likely that an increase in SUR1 may play an important role in preventing mitochondrial dysfunction in old rats or at least partially restoring their function to the level of adult rats. Because it is well known about the protective role of mitochondrial K_ATP_ channels, an important part of which is the SUR1 receptor, against various pathological factors, in particular ischemia. Activation of these membrane channels prevents mitochondrial calcium overload and, as noted above, prevents mPTP opening and prevents cell apoptosis [24, 30, 31]. It is likely that not only K_ATP_ channels [29] but even to a greater extent that GSH may be involved in the inhibition of mPTP opening. The mitochondrial respiratory chain is a potent source of reactive oxygen species, which are inducers of mPTP opening. Antioxidant enzymes function in the mitochondria to maintain the redox balance, including GSH, which can reduce H_2_O_2_ and lipid hydroperoxides with the help of glutathione peroxidases (GPx1, GPx4). In general, GSH can maintain mitochondrial redox balance through reverse posttranslational modifications of proteins, particularly the Krebs cycle and the electron transport chain, through S-glutathionylation/deglutathionylation [3]. Thus, inhibition of mPTP opening may be a consequence of a significant reduction in oxidative stress, which was significantly lower in GSH-treated animals.

In contrast to SUR1, the expression of SUR2 receptors in old rats remained practically unchanged when glutathione was administered. But if SUR1 and SUR2 are part of different subtypes of K_ATP_ channels located on mitochondrial and sarcolemmal membranes, respectively, then, the function of these channels is the same. This is a protective function in the case of a critical decrease in the energy reserves of the cell, namely, ATP. However, K_ATP_ channels of the inner mitochondrial membrane control mitochondrial function through membrane depolarization, while K_ATP_ channels of the cytoplasmic membrane through membrane hyperpolarization control the function of cells and even systems. They regulate the tone of blood vessels, contractile activity of the heart and metabolism. But both of these processes, depolarization of the mitochondrial membrane and hyperpolarization of the plasma membrane, inhibit the entry of calcium, in the first case into the mitochondrial matrix and in the second case into the cytoplasm of the cell. This suppresses the metabolism and contractile work of muscle cells in conditions of energy deficit and thereby ensures their preservation under adverse conditions, in particular, at ischemia. However, in the absence of reliable changes in SUR2 expression when glutathione is administered to old rats, other mechanisms are probably involved in cardioprotection, possibly the antioxidant effect of glutathione, possibly an increase in hydrogen sulfide levels due to glutathione administration [32]. And perhaps, this cardioprotection occurs due to increased expression of Kir6.2 subunits of cardio-specific K_ATP_ channels. As for the possible protective value of increased expression of pore-forming Kir6.2 subunits of K_ATP_ channels in the myocardium, it is considered not as important in the protection of the myocardium during ischemia as changes in the expression of the regulatory SUR2A subunit [27, 33]. It is believed that Kir6.2 subunits of K_ATP_ channels are in excess in the myocardium, and for the formation of a larger number of cardio-specific SUR2A/Kir6.2 K_ATP_ channels, an increase mainly in SUR2A subunits is necessary [33]. However, there are also studies that have shown raised cardiac protection due to increased expression of Kir6.2 [34].

However, the mechanism by which glutathione leads to the upregulation expression of some subunits of K_ATP_ channels remains unstudied. It is likely that one of the variants of the regulation of the expression of K_ATP_ channels can be feedback in the regulation. According to some studies, reduced glutathione can inhibit the activity of K_ATP_ channels, in particular through their S-glutathionylation [13, 35]. Therefore, the introduction of exogenous reduced glutathione can cause a partial decrease in the activity of the K_ATP_ channel system, which can probably cause the strengthening expression of K_ATP_ channels according to the principle of feedback, and an increase in their density on cell membranes.

Undoubtedly, the data on the increased regulation of the expression of K_ATP_ channels in old rats upon the introduction of exogenous glutathione can be considered a novelty of this study. At the same time, it was shown for the first time that such an increase in the expression of certain subunits of K_ATP_ channels is accompanied by an increase in the relaxation responses of vascular rings of the aorta on stimulation of K_ATP_ channels and inhibition of mPTP opening. Despite the fact that the limitation of this work is the lack of research on the expression of K_ATP_ channels at the protein level, the effects of openers and inhibitors of these channels in functional experiments on isolated aortic rings and suspension of isolated mitochondria fully support the idea of an increase in the membrane density (number) of K_ATP_ channels.

## 5. Conclusions

Thus, the administration of exogenous glutathione significantly increased the content of the reduced form of glutathione in the heart tissue and reduced the indicators of oxidative stress in the heart mitochondria of old rats, which increased with age. Simultaneously, mPTP opening was inhibited, probably due to a decrease in sensitivity to calcium ions, and vasorelaxation responses of isolated aortic rings to the introduction of the K_ATP_ channel openers flocalin and pinacidil were enhanced. All of these protective changes with glutathione administration were associated with increased expression of Kir6.1, Kir6.2, and SUR1 subunits of K_ATP_ channels.

## Figures and Tables

**Table 1:** The primer sequences that were used to determine gene expression.

Gene	Product size	Accession number	Primer sequence
KCNJ8 (Kir6.1)	94 bp	NM_017099.4	Up 5′-TCTCTTCTCCATCGAGGTTCA-3′ Dw 5′-CTGCAGAATCAAAACCGTGAT-3′
KCNJ11 (Kir6.2)	96 bp	NM_031358.3	Up 5′-ATGAGAGAAAGGGGGACAAGA-3′ Dw 5′-AGGCTGGAGTCAAGGGTAGAG-3′
ABCC8 (SUR1)	101 bp	L40624.1	Up 5′-GGGCTTCTGGTGATCCTCTAC-3′ Dw 5′-GGCTTTACTTCCCTTGGTGTC-3′
ABCC9 (SUR2)	99 bp	D83598.1	Up 5′-GCTCTGGAAATTGCTCAGTTG-3′ Dw 5′-CTGTCCAACGCTGAAGTTCTC-3′
ACTB	97 bp	V01217.1	Up 5′-AAGTCCCTCACCCTCCCAAAA-3′ Dw 5′-AAGCAATGCTGTCACCTTCCC-3′

**Table 2:** Markers of oxidative stress in heart mitochondria and levels of total, oxidized, and reduced glutathione in heart tissue.

	Adult	Old	Old + glutathione
^•^O_2_^−^ (nmol/min/mg protein)	4.43 ± 0.20	17.16 ± 4.63^∗^	6.22 ± 0.34^∗^^,#^
H_2_O_2_ (pmol/mg protein)	12.86 ± 3.43	24.47 ± 3.28^∗^	11.49 ± 0.15^#^
MDA (nmol/mg protein)	2.29 ± 0.17	5.76 ± 0.34^∗^	3.66 ± 0.19^#^
DC (ng/mg protein)	3.78 ± 0.25	16.21 ± 2.55^∗^	7.84 ± 1.88^#^
Total GSH (nmol/mg tissue)	1026.77 ± 38.02	993.9 ± 34.69	1394.33 ± 73.21^∗^^,#^
GSSG (nmol/mg tissue)	175.27 ± 10.16	209.91 ± 20.60	229.71 ± 21.40
GSH (nmol/mg tissue)	676.24 ± 23.29	574.13 ± 25.48^∗^	934.90 ± 55.96^∗^^,#^

Note: ^•^O_2_^−^: superoxide; MDA: malondialdehyde; DC: diene conjugates; total GSH: total glutathione; GSSG: oxidized glutathione; GSH: reduced glutathione; data are represented as **mean** ± **SEM**. In experiments, to determine markers of oxidative stress for all groups of animals, there were **n** = 12. Glutathione levels were measured in adult and old glutathione-treated rats (**n** = 7) and old rats (**n** = 8). ^∗^*Р* < 0.05 versus adult; ^#^**P** < 0.05 versus old.

## Data Availability

The data that support the findings of this study are available in the methods and results of this article. The data are available upon request to the corresponding author.
